# Peripheral immune landscape and natural killer-like B cells in human Vogt-Koyanagi-Harada disease

**DOI:** 10.1093/lifemedi/lnac047

**Published:** 2022-12-08

**Authors:** He Li, Lei Zhu, Xiuxing Liu, Lihui Xie, Rong Wang, Zhaohuai Li, Zhaohao Huang, Shizhao Yang, Binyao Chen, Jinguo Ye, Yingfeng Zheng, Wenru Su

**Affiliations:** State Key Laboratory of Ophthalmology, Zhongshan Ophthalmic Center, Sun Yat-sen University, Guangdong Provincial Key Laboratory of Ophthalmology and Visual Science, Guangzhou 510060, China; State Key Laboratory of Ophthalmology, Zhongshan Ophthalmic Center, Sun Yat-sen University, Guangdong Provincial Key Laboratory of Ophthalmology and Visual Science, Guangzhou 510060, China; State Key Laboratory of Ophthalmology, Zhongshan Ophthalmic Center, Sun Yat-sen University, Guangdong Provincial Key Laboratory of Ophthalmology and Visual Science, Guangzhou 510060, China; State Key Laboratory of Ophthalmology, Zhongshan Ophthalmic Center, Sun Yat-sen University, Guangdong Provincial Key Laboratory of Ophthalmology and Visual Science, Guangzhou 510060, China; State Key Laboratory of Ophthalmology, Zhongshan Ophthalmic Center, Sun Yat-sen University, Guangdong Provincial Key Laboratory of Ophthalmology and Visual Science, Guangzhou 510060, China; State Key Laboratory of Ophthalmology, Zhongshan Ophthalmic Center, Sun Yat-sen University, Guangdong Provincial Key Laboratory of Ophthalmology and Visual Science, Guangzhou 510060, China; State Key Laboratory of Ophthalmology, Zhongshan Ophthalmic Center, Sun Yat-sen University, Guangdong Provincial Key Laboratory of Ophthalmology and Visual Science, Guangzhou 510060, China; State Key Laboratory of Ophthalmology, Zhongshan Ophthalmic Center, Sun Yat-sen University, Guangdong Provincial Key Laboratory of Ophthalmology and Visual Science, Guangzhou 510060, China; State Key Laboratory of Ophthalmology, Zhongshan Ophthalmic Center, Sun Yat-sen University, Guangdong Provincial Key Laboratory of Ophthalmology and Visual Science, Guangzhou 510060, China; State Key Laboratory of Ophthalmology, Zhongshan Ophthalmic Center, Sun Yat-sen University, Guangdong Provincial Key Laboratory of Ophthalmology and Visual Science, Guangzhou 510060, China; State Key Laboratory of Ophthalmology, Zhongshan Ophthalmic Center, Sun Yat-sen University, Guangdong Provincial Key Laboratory of Ophthalmology and Visual Science, Guangzhou 510060, China; State Key Laboratory of Ophthalmology, Zhongshan Ophthalmic Center, Sun Yat-sen University, Guangdong Provincial Key Laboratory of Ophthalmology and Visual Science, Guangzhou 510060, China

**Keywords:** Vogt-Koyanagi-Harada disease, single-cell RNA sequencing, natural killer-like B cells, autoimmunity

## Abstract

Vogt-Koyanagi-Harada (VKH) disease is a systemic autoimmune disorder threatening the eyesight. The pathogenic mechanisms and biomarkers reflecting disease severity and predicting treatment response require further exploration. Here, we performed a single-cell analysis of peripheral blood mononuclear cells (PBMC) obtained from eight patients with VKH disease and eight healthy controls to comprehensively delineate the changes in VKH disease. We showed a mixture of inflammation, effector, and exhausted states for PBMCs in VKH disease. Notably, our study implicated a newly identified B cell subset, natural killer-like B cells (K-BC) characterized by expressing CD19 and CD56, was correlated with VKH disease. K-BCs expanded in VKH disease, fell back after effective treatment, and promoted the differentiation of pathogenic T cells. Overall, we mapped the peripheral immune cell atlas in VKH disease and indicated the pathogenic role and potential value in predicting treatment response of K-BCs.

## Graphical Abstract

**Graphical Abstract:**
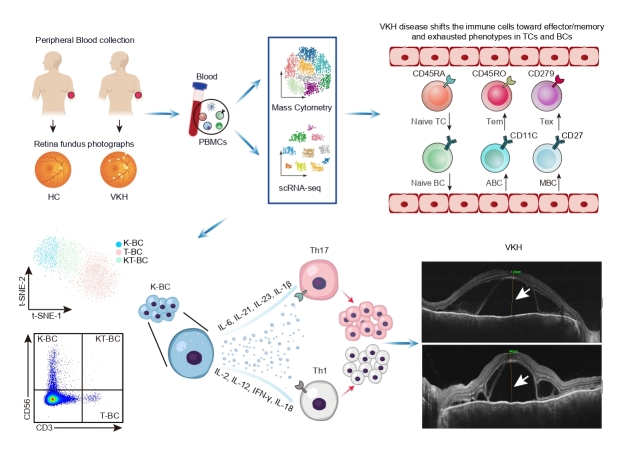

## Introduction

Vogt-Koyanagi-Harada (VKH) disease is one of the major sight-threatening types of uveitis in Asia with a high risk of blindness [1–3]. It is a systemic refractory autoimmune disorder characterized by bilateral granulomatous pan-uveitis and is frequently accompanied by multiple systemic symptoms, including leukoderma, paratrichosis, and neurological and auditory manifestations [1, 4]. The diagnosis of VKH disease mainly relies on its clinical features [5]; however, there are no disease-specific biomarkers to assist in diagnosis. Rapid and aggressive systemic corticosteroids combined with immunosuppressive drugs and/or biological agents are the major treatments for VKH disease [6]. However, untoward side effects, such as hirsutism and infection, may occur with the use of systemic corticosteroids and immunosuppressive drugs [7, 8]. Additionally, the high cost of biological agents often limits their use in uveitis [9]. Based on current studies, genetic [3, 10], and environmental factors, such as infections [11] are associated with VKH disease pathogenesis. The autoimmune response is thought to be a major contributor to VKH disease [1, 12]; however, the specific cell types/subtypes and molecules still await further research.

CD4^+^ effector T cells (TC), especially T helper (Th)-1 and Th17 cells are thought to be critical participants in uveitis pathogenesis [13, 14]. During VKH, a hyperactive response of Th17 and Th1 cells are considered to promote disease development and these cells have been found to accumulate in the blood, aqueous fluid, and other tissues of VKH patients [15–17]. In animal experiments, adoptive transfer of autoreactive CD4^+^ T cells can induce uveitis in naïve mice [18]. In addition to TCs, recent studies have also demonstrated the relevance of various immune cells to VKH disease [10]. By analyzing circulating monocytes (MC), a disease-associated proinflammatory MC subtype that highly expresses ISG15 was discovered in patients with VKH disease [19]. Additionally, B cell (BC)-related molecules, B cell activating factor (BAFF), B cell chemoattractant, and autoantibodies have also been detected in aqueous humor samples [20–22]. However, previous approaches were largely restricted to well-established antibody panels or sorted cell samples based on prior knowledge, and therefore preclude the comprehensive characterization of immune dysfunction and identification of novel pathogenic cell populations and pathways in VKH disease. Thus, it is desirable to establish a comprehensive immune atlas to obtain a deeper understanding of the pathological mechanisms of VKH disease.

Single-cell analysis tools are a group of advanced technologies that enable high-throughput analysis of thousands of individual cells, providing information about the transcriptome and proteomics [23]. In recent years, utilizing these powerful tools, the pathogenesis of a variety of diseases has been revealed [24–26]. Meanwhile, novel cell types that break the traditional cell classification emerge in an endless stream, and these cells may play important roles in diseases [27–29]. Single-cell analysis of human peripheral blood mononuclear cells (PBMC) has also exhibited a wide range of applications, such as delineating the peripheral immune states in COVID-19 [30] and predicting drug response in patients with chronic myeloid leukemia [31]. In this study, we combined single-cell RNA sequencing (scRNA-seq) and mass cytometry (CyTOF), to develop a comprehensive peripheral immune landscape of VKH disease. High-throughput analysis using these tools provides reciprocal supporting data at transcriptional and protein levels. The unbiased clustering function of single-cell analysis tools allowed us to recognize a newly identified BC subset, natural killer (NK)-like BCs (K-BC) which were characterized by the co-expression of CD56 and CD19, markers of NKs and BCs. Our study provides evidence for the relationship between K-BCs and VKH disease pathogenesis and the potential for K-BCs to act as indicators for predicting disease severity and treatment response.

## Results

### Study design and cell type identification of PBMCs

**Figure 1.**
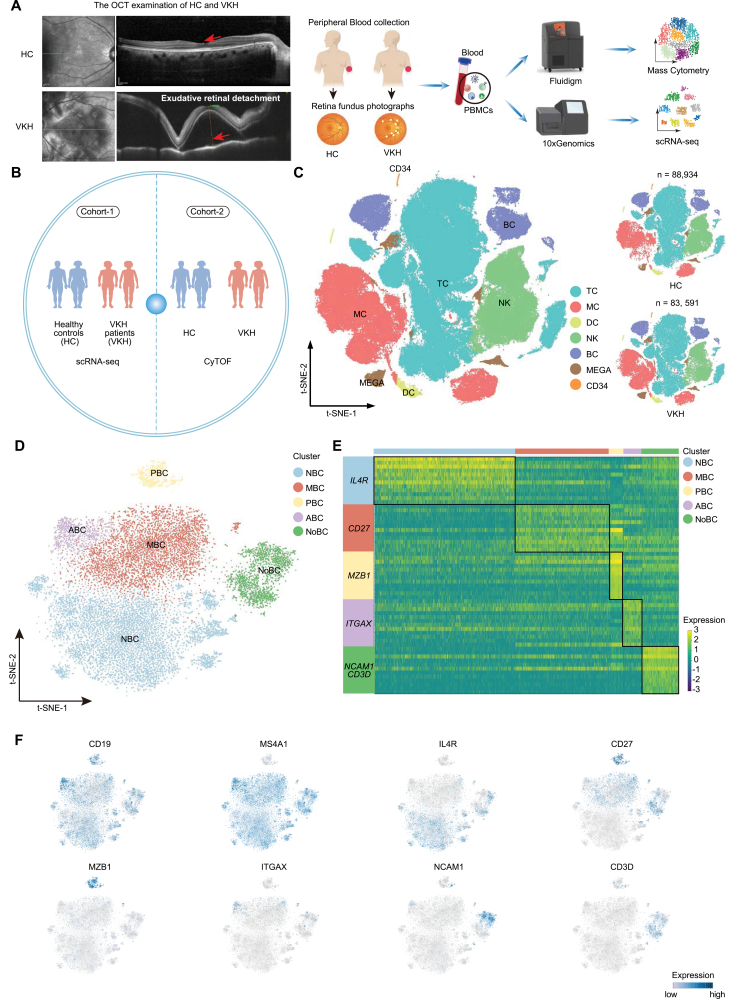
**Study design and cell type identification of PBMCs.** (A) Schematic diagram of the experimental design. (B) Schematic representation of study datasets (C) *t*-Distributed stochastic neighbor embedding (*t*-SNE) clustering of PBMCs of HC and VKH originated from scRNA-seq data. (D) *t*-SNE plots of B cell subsets originated from scRNA-seq data. (E) Heatmap showing scaled expression of discriminative gene sets for B cell subsets originated from scRNA-seq data. (F) *t*-SNE plots of canonical markers for B cell subsets originated from scRNA-seq data.

To comprehensively demonstrate the peripheral immune changes associated with VKH disease, we divided patients with VKH disease (VKH) and healthy controls (HC) into two cohorts. Blood was collected from both groups, and PBMCs were isolated for further analysis (Fig. 1A and 1B). In cohort-1, we conducted scRNA-seq to create a comparative framework detailing the influence of VKH disease on immune cell functions at the transcriptional level (Fig. 1B). Additionally, we performed CyTOF analysis for cohort-2 to reveal immune cell distribution at the protein level (Fig. 1B).

PBMCs were converted into barcoded scRNA-seq libraries using 10× Genomics for downstream analysis. We combined CellRanger software with the Seurat package for initial processing, quality control, and analysis of the sequencing data. After quality control, 88,934 and 83,591 high-quality cells were obtained from the HC and VKH groups, respectively (Fig. 1C). According to classical markers and other unique genes, we identified CD34^+^ progenitor cells, megakaryocytes, and five major immune cell types, including TCs, NKs, BCs, MCs, and dendritic cells (DC) (Figs. 1C and S1). Considering the heterogeneity within immune cell types, we further re-clustered the major immune cell types. Corresponding to known peripheral blood immune cell subsets, TCs were divided into CD4^+^, CD8^+^, CD4^−^CD8^−^ double negative, CD4^+^CD8^+^ double positive, and proliferation TCs (Pro-T) (Fig. S2A−S2C). CD4^+^ TCs were subdivided into naïve (CD4 naïve), central memory (CD4 Tcm), effector memory (CD4 Tem), regulatory (CD4 Treg), cytotoxic (CD4 CTL), and exhausted (CD4 Tex) CD4^+^ TCs (Fig. S2D−S2F). CD8^+^ TCs were subdivided into naïve (CD8 Naïve), effector memory (CD8 Tem), cytotoxic (CD8 CTL), and exhausted (CD8 Tex) CD8^+^ TCs (Fig. S2G–S2I). NKs were divided into three subclusters incorporating NK1, NK2, and NK3, based on the expression of NCAM1, B3GAT1, and FCGR3A (Fig. S2J–S2L). We also identified myeloid cell (MCs and DCs) subsets according to classic markers. MCs were subdivided into classical (CD14 MC), non-classical (CD16 MC), and intermediate (Inter MC) MCs (Fig. S2M–S2O). We also re-clustered DCs into conventional DC1(cDC1), conventional DC2 (cDC2), and plasmacytoid DC (pDC) (Fig. S2P–S2R). Unbiased clustering of BCs revealed four conventional BC subsets including naïve BCs (NBC), memory BCs (MBC), plasma cells (PBC), and age-associated BCs (ABC) (Fig. 1D–1F). Interestingly, a novel BC subset (NoBC) was also revealed (Fig. 1D–1F). NoBCs were different from the conventional BC subsets and were characterized by high expression of CD19, a BC marker, along with NCAM1 and CD3D, the markers for TCs and NKs respectively (Fig. 1E and 1F).

To prove the existence of these clusters in the scRNA-seq data, we evaluated PBMCs at protein level. CyTOF is a next generation flow cytometry platform that enables simultaneous detection of up to 100 metal-conjugated antibodies on a single cell [32]. Utilizing this tool, the corresponding major immune cells (Fig. S3A and S3B) and their subsets, including 13 TC subsets (Fig. S3C and S3D), three NK cell subsets (Fig. S4A and S4B), five myeloid subsets (Fig. S4C and S4D), and five BC subsets (Fig. S4E and S4F), were identified based on their markers. The NoBC subset with relatively high expression of CD3 and CD56 in BCs was also observed by CyTOF (Fig. S4E and S4F).

Collectively, our scRNA-seq and CyTOF data incorporated the majority of known cell types and immune cell subsets among PBMCs. Notably, we identified a novel BC subset, NoBC, which was not included in the classical immune cell classification.

### Mapping immune cell atlas in VKH disease

**Figure 2.**
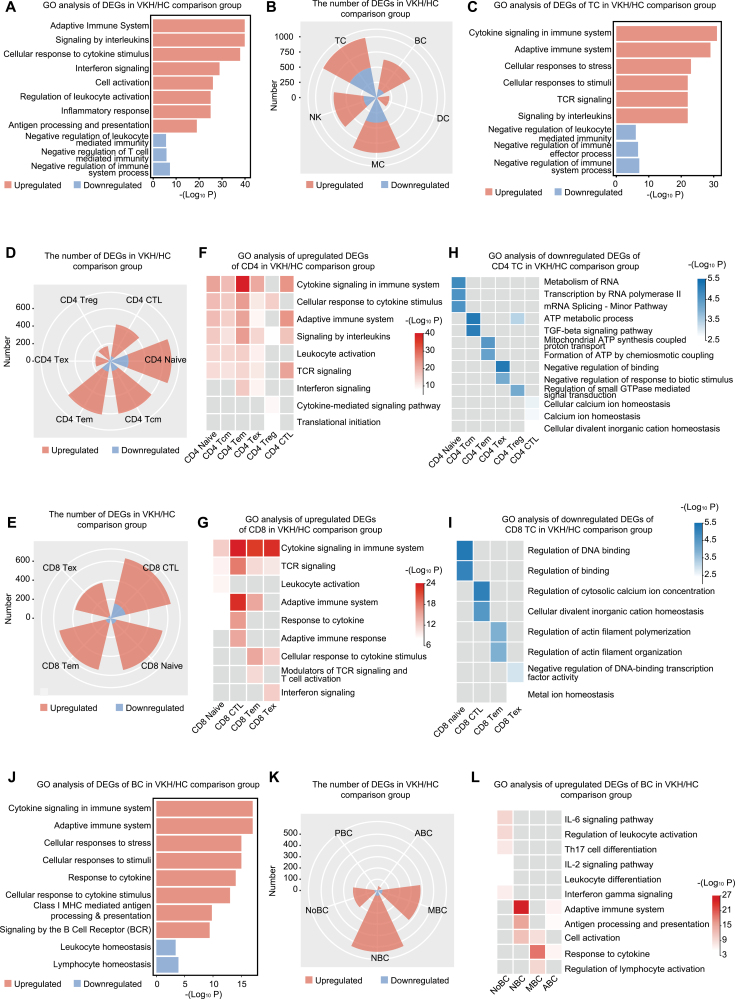
**Mapping immune cell atlas in VKH disease compared to HC.** (A) Heatmap showing representative GO terms enriched in up- or down-regulated DEGs of total PBMCs in the VKH/HC comparison group. (B) Rose diagrams showing the numbers of up- and down-regulated DEGs of major immune cell types in the VKH/HC comparison group. (C) Heatmap showing representative GO terms enriched in up- or down-regulated DEGs of T cells in the VKH/HC comparison group. (D) Rose diagrams showing the numbers of up- and down-regulated DEGs of CD4^+^ T cell subsets in the VKH/HC comparison group. (E) Rose diagrams showing the numbers of up- and down-regulated DEGs of CD8^+^ T cell subsets in the VKH/HC comparison group. (F) Heatmap showing representative GO terms enriched in upregulated DEGs of CD4^+^ T cell subsets in the VKH/HC comparison group. (G) Heatmap showing representative GO terms enriched in upregulated DEGs of CD8^+^ T cell subsets in the VKH/HC comparison group. (H) Heatmap showing representative GO terms enriched in down-regulated DEGs of CD4^+^ T cell subsets in the VKH/HC comparison group. (I) Heatmap showing representative GO terms enriched in down-regulated DEGs of CD8^+^ T cell subsets in the VKH/HC comparison group. (J) Heatmap showing representative GO terms enriched in up- or down-regulated DEGs of B cells in the VKH/HC comparison group. (K) Rose diagrams showing the numbers of up- and down-regulated DEGs of B cell subsets in the VKH/HC comparison group. (L) Heatmap showing representative GO terms enriched in upregulated DEGs of B cell subsets in the VKH/HC comparison group.

To demonstrate the transcriptional signatures of the peripheral immune system in VKH disease, we conducted the differentially expressed gene (DEG) analysis of immune cell types between VKH and HC groups using scRNA-seq data. Subsequent gene ontology (GO) analysis was performed to identify the biological significance of the DEGs. We first explored the general transcriptional changes in VKH disease by evaluating total PBMCs. GO analysis of DEGs in total PBMCs revealed that genes included in pathways related to inflammation (encompassing signaling by interleukins, interferon signaling, and inflammatory response), cell activation (encompassing cell activation and regulation of leukocyte activation), and adaptive immune response (encompassing adaptive immune system and antigen processing and presentation) were upregulated (Fig. 2A). Whereas those included in pathways associated with negative regulation of immunity were downregulated (Fig. 2A).

We then delineated VKH disease-associated changes in cell type-specific transcriptomes. Among the five major immune cell types, VKH disease-induced gene expression changes were mainly upregulated, among which TCs showed the highest and DCs showed the lowest number of DEGs (Fig. 2B). The upregulated DEGs of TCs were enriched in inflammation-related pathways (encompassing cytokine signaling in immune system and signaling by interleukins) and TC response-related pathways (annotated as adaptive immune system and cellular responses to stimuli) (Fig. 2C). The down-regulated DEGs of TCs were enriched in pathways related to negative regulation of immunity (Fig. 2C). Similarly, in TC subsets, upregulated DEGs were also outnumbered compared to down-regulated DEGs (Fig. 2D and 2E). In both CD4^+^ and CD8^+^ TCs, the upregulated DEGs are involved in inflammation-related pathways (such as cytokine signaling in immune system and interferon signaling), and leukocyte activation pathways (Fig. 2F and 2G). Additionally, by performing GO analysis on the down-regulated DEGs of TC subsets, we observed metabolism of RNA, TGF-beta signaling pathway, and ATP related pathways were enriched in CD4^+^ TC subsets, while regulation of DNA binding, cytosolic calcium ion concentration, and actin filament polymerization pathways were enriched in CD8 TC^+^ subsets (Fig. 2H and 2I).

Regarding BCs, the upregulated DEGs are involved in B cell response-related pathways (annotated as signaling by the B cell receptor) and inflammation-related pathways (annotated as cytokine signaling in immune system and response to cytokine) (Fig. 2J). DEGs involved in the class I MHC mediated antigen processing and presentation pathway were also upregulated in BCs, indicating the enhanced antigen-presenting by BCs in VKH disease (Fig. 2J). We further conducted DEG analysis on BC subsets. VKH disease-induced gene expression changes in BC subsets were also mainly upregulated (Fig. 2K). Among the BC subsets, NBCs showed the highest number of DEGs, and the number of DEGs in MBCs and NoBCs was also evident (Fig. 2K). Subsequent GO analysis revealed that the upregulated DEGs in NBCs, MBCs, and ABCs were mainly enriched in pathways related to antigen processing and presentation, cell activation, and response to cytokine (Fig. 2L). Interestingly, the pathways enriched in NoBCs were mainly related to cytokine secretion (incorporating IL-6, IL-2, and interferon-gamma signaling pathways) and cell differentiation (annotated as Th17 cell differentiation and leukocyte differentiation) (Fig. 2L). As for down-regulated genes of BC subsets in VKH disease, GO analysis showed down-regulated genes were involved in generation of precursor metabolites and energy, ATP metabolic process, lymphocyte homeostasis, and leukocyte homeostasis (Fig. S5A).

Regarding innate immune cells, MCs showed evident upregulation of genes involved in inflammation-related pathways (encompassing inflammatory response, interferon, and interleukin-1 family signaling pathways) compared to DCs and NKs (Fig. S5B). The genes involved in tumor necrosis factor (TNF) signaling pathway and positive regulation of leukocyte activation pathway were upregulated in DCs, whereas NKs showed an evident response to stimuli and cytokines (Fig. S5B). Moreover, genes involved in negative regulation of cell killing and leukocyte-mediated cytotoxicity pathways were downregulated in NKs, indicating increased cytotoxicity of these cells in VKH disease (Fig. S5C). We further conducted DEG analysis on MC, NK, and DC subsets. Evident upregulation of genes involved in inflammation-related pathways was also shown in these innate immune cell subsets, especially in MC subsets (Fig. S5D–S5F). Additionally, antigen processing and presentation-related pathways were upregulated in MC subsets and cDC2 (Fig. S5E and S5F).

These results indicate an inflammatory and activated state of the peripheral immune system in VKH disease compared to HCs. In addition, the NoBC transcriptome indicated upregulated cytokine production and T cell differentiation pathways in VKH disease.

### VKH disease shifts the immune cells toward effector/memory and exhausted phenotypes in TCs and BCs

**Figure 3.**
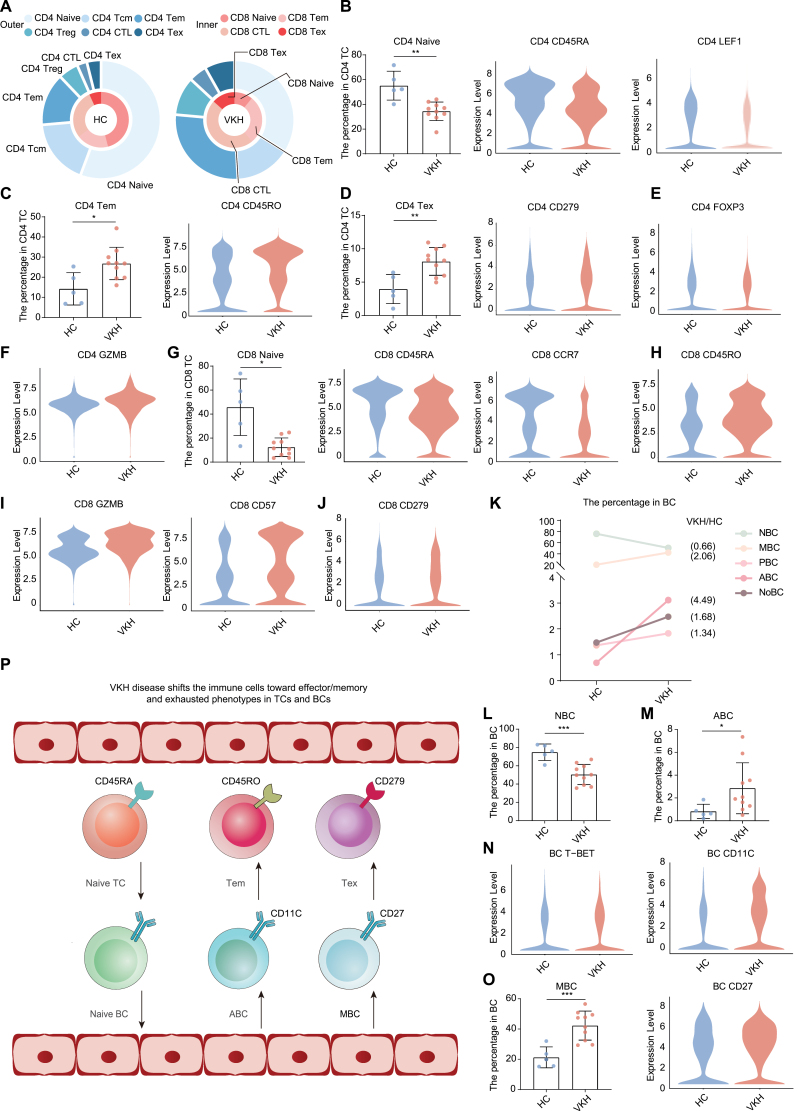
**VKH disease shifts the cellular composition toward effector/memory and exhausted phenotypes.** (A) Pie charts showing relative cluster abundance of T cell subsets in the HC and VKH groups originated from CyTOF data. (B) Bar chat shows percentage of CD4 naïve in CD4 TC from HC (*n* = 10) and VKH (*n* = 5) group originated from CyTOF data. The values represent the mean ± SD. Significance was determined using unpaired Student’s *t-*test. **P < 0.01. The violin plots of expression of CD45RA and LEF1 in CD4 TC from HC and VKH groups originated from CyTOF data. (C) Bar chat shows percentage of CD4 Tem in CD4 TC from HC (*n* = 10) and VKH (*n* = 5) group originated from CyTOF data. The values represent the mean ± SD. Significance was determined using unpaired Student’s *t*-test. *P < 0.05. The violin plot of expression of CD45RO in CD4 TC from HC and VKH groups originated from CyTOF data. (D) Bar chat shows percentage of CD4 Tex in CD4 TC from HC (*n* = 10) and VKH (*n* = 5) group originated from CyTOF data. The values represent the mean ± SD. Significance was determined using unpaired Student’s *t*-test. **P < 0.01. The violin plot of expression of CD279 in CD4 TC from HC and VKH groups originated from CyTOF data. (E) The violin plot of expression of FOXP3 in CD4 TC from HC and VKH groups originated from CyTOF data. (F) The violin plot of expression of GZMB in CD4 TC from HC and VKH groups originated from CyTOF data. (G) Bar chat shows percentage of CD8 naïve in CD8 TC from HC (*n* = 10) and VKH (*n* = 5) group originated from CyTOF data. The values represent the mean ± SD. Significance was determined using unpaired Student’s *t*-test. **P < 0.01. The violin plots of expression of CD45RA and CCR7 in CD8 TC from HC and VKH groups originated from CyTOF data. (H) The violin plot of expression of CD45RO in CD8 TC from HC and VKH groups originated from CyTOF data. (I) The violin plots of expression of GZMB and CD57 in CD8 TC from HC and VKH groups originated from CyTOF data. (J) The violin plot of expression of CD279 in CD8 TC from HC and VKH groups originated from CyTOF data. (K) Line chart shows percentage of B cell subsets in BC from HC and VKH groups originated from CyTOF data. VKH/HC shows that the fold change of cell ratio in B cell subsets in the VKH/HC comparison group. (L) Bar chat shows percentage of NBC in BC from HC (*n* = 10) and VKH (*n* = 5) group originated from CyTOF data. The values represent the mean ± SD. Significance was determined using unpaired Student’s *t-*test. ***P < 0.001. (M) Bar chat shows percentage of ABC in BC from HC (*n* = 10) and VKH (*n* = 5) group originated from CyTOF data. The values represent the mean ± SD. Significance was determined using unpaired Student’s *t-*test. *P < 0.05. (N) The violin plots of expression of T-BET and CD11C in BC from HC and VKH groups originated from CyTOF data. (O) Bar chat shows percentage of MBC in BC from HC (*n* = 10) and VKH (*n* = 5) group originated from CyTOF data. The values represent the mean ± SD. Significance was determined using unpaired Student’s *t-*test. ***P < 0.001. The violin plots of expression of CD27 in BC from HC and VKH groups originated from CyTOF data. (P) Schematic illustration of the immune changing between VKH and HC groups.

To delineate the changes in immune cell composition in VKH disease, we referred to the CyTOF data. Although there was no statistically significant difference in the percentage of major immune cell types between the HC and VKH groups (Fig. S6A–S6D), the composition of CD4^+^ and CD8^+^ TCs was significantly changed in VKH disease (Fig. 3A). In CD4^+^ TCs, the percentage of naïve TCs and the expression of the related markers (CD45RA and LEF1) decreased, while the percentages of Tem and Tex and the expression of the corresponding markers (CD45RO and CD279, respectively) increased in VKH disease (Fig. 3B–3D). Although the percentage of Tcm, Treg, and CTL was not significantly changed (Fig. S6E–S6G), reduced expression of the Treg marker (FOXP3) and increased expression of the CTL marker (GZMB) were observed in VKH disease (Fig. 3E and 3F). In CD8^+^ TCs, the percentage of naïve TCs and the expression of their markers (CD45RA and CCR7) were also decreased (Fig. 3G). Additionally, the expression of the Tem marker (CD45RO), CTL markers (GZMB and CD57), and the Tex marker (CD279) in CD8^+^ TCs increased in VKH disease (Fig. 3H–J), although no significant changes were detected in the percentage of the above CD8^+^ TC subsets (Fig. S6H–S6J).

Regarding BCs, similar shifts from the naïve to the effector or memory phenotype were observed (Fig. 3K). There was a lower percentage of NBCs in the VKH group (Fig. 3L). In addition, the percentage of ABCs and MBCs, and the expression of their corresponding markers (T-BET, CD11C, and CD27) increased (Fig. 3M–3O). Although the percentages of PBCs and NoBCs were not significantly changed (Fig. S6K and S6L), the proportion of NoBCs increased by 1.68 folds in VKH disease (Fig. 3K). As for innate immune cell subsets, their percentage within total PBMCs did not change significantly, except for the NK3 subset (Fig. S6M–S6T).

These results demonstrated that VKH disease shifted the peripheral TCs and BCs from a naive phenotype to effector/memory and exhausted phenotypes in the perspective of cell composition (Fig. 3P).

### Heterogeneity of NoBCs

**Figure 4.**
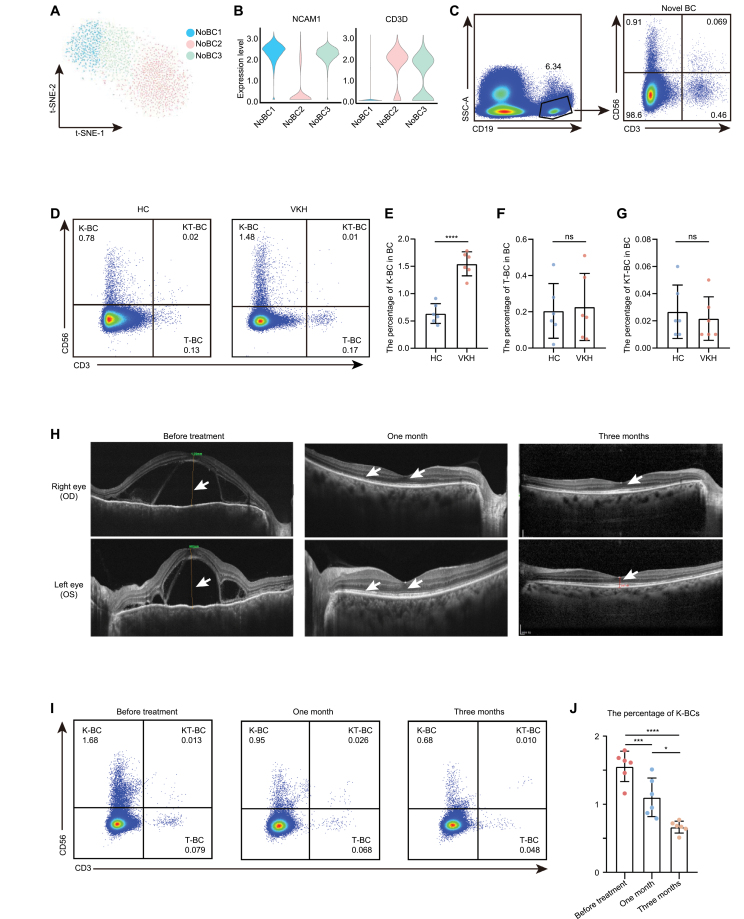
**K-BC correlated to VKH disease.** (A) *t*-SNE plots of NoBC subsets originated from scRNA-seq data. (B) The violin plot of expression of NCAM1 and CD3D in NoBC subsets originated from scRNA-seq data. (C) The expression of CD19, CD56 and CD3 in NoBC were measured by flow cytometry. (D–G) The percentage of K-BC, T-BC, and KT-BC in BC from HC and VKH group was measured by flow cytometry (D) and quantified (E–G). Each group contains six samples. The values represent the mean ± SD. Significance was determined using unpaired Student’s *t*-test. ****P < 0.0001. ns, not significant. (H) The OCT examination of VKH patient in different stages (before treatment, after treatment for 1 month, and after treatment for 3 months). The white arrow showed the process of absorption of subretinal fluid before and after treatment. OD: Right eye. OS: Left eye. (I and J) The percentage of K-BC in BC from VKH patient in different stages (before treatment, after treatment for 1 month, and after treatment for 3 months) was measured by flow cytometry (I) and quantified (J). Each group contains six samples. The values represent the mean ± SD. Significance was determined using ANOVA. *P < 0.05, ***P < 0.001, ****P < 0.0001.

In our scRNA-seq and CyTOF data, NoBCs were recognized and showed an increasing trend in VKH disease, which aroused our interest. Thus, we explored this group of cells in more depth. We performed unbiased clustering in NoBCs and identified three clusters which could be distinguished by their expression level of CD3D and NCAM1 (Fig. 4A and 4B). CD3D encodes subunit of CD3 and NCAM1 encodes CD56. Utilizing antibodies of CD3, CD56, and CD19, three clusters of NoBCs were also identified by flow cytometry including NoBC1 (CD19^+^ CD56^+^ NoBCs), NoBC2 (CD19^+^ CD3^+^ NoBCs), and NoBC3 (CD19^+^ CD56^+^ CD3^+^ NoBCs) (Figs. S6U and 4C). The phenotype of NoBC1 is analogous to that of a newly identified natural killer-like BCs that expresses markers of BC and NK and exists in both human and mouse [33–36]. Thus, NoBC1 were annotated as natural killer-like B cells (K-BC). The phenotype of NoBC2 was similar to that of previously reported TB cells co-expressing TCR and BCR [37]. Thus, NoBC2 were annotated as T-BC. The NoBC3 subset did not share a similar phenotype with any previously reported PBMC cell subsets. Based on the co-expression of BC, TC, and NK markers on NoBC3, we named these cells natural killer-like T-BCs (KT-BC).

### K-BCs are correlated with VKH disease

To further identify the relationship between NoBC subsets and VKH disease, we evaluated the changes in their proportions in VKH disease using flow cytometry. The percentage of K-BCs was significantly higher in patients with VKH disease than that in HCs, while the percentage of T-BCs and KT-BCs showed no significant changes (Fig. 4D–4G). K-BCs have been reported to present immunomodulatory property in the eradication of microbial infection, and whether K-BCs involve in VKH disease is unclear. To further explore the relationship between K-BCs and the clinical course of VKH disease, we evaluated the changes in the proportions of K-BCs, T-BCs, and KT-BCs in patients with VKH disease, who had received immunosuppressive therapy (Fig. 4H–4J). Intriguingly, only the percentage of K-BCs decreased after treatment and disease improvement which was measured using optical coherence tomography (OCT), a commonly used ocular fundus examination (Figs. 4I, 4J, S7A and S7B). These results suggested that K-BCs might correlate with VKH disease and serve as an indicator of disease severity and treatment response.

### K-BCs promote the differentiation of pathogenic TCs

**Figure 5.**
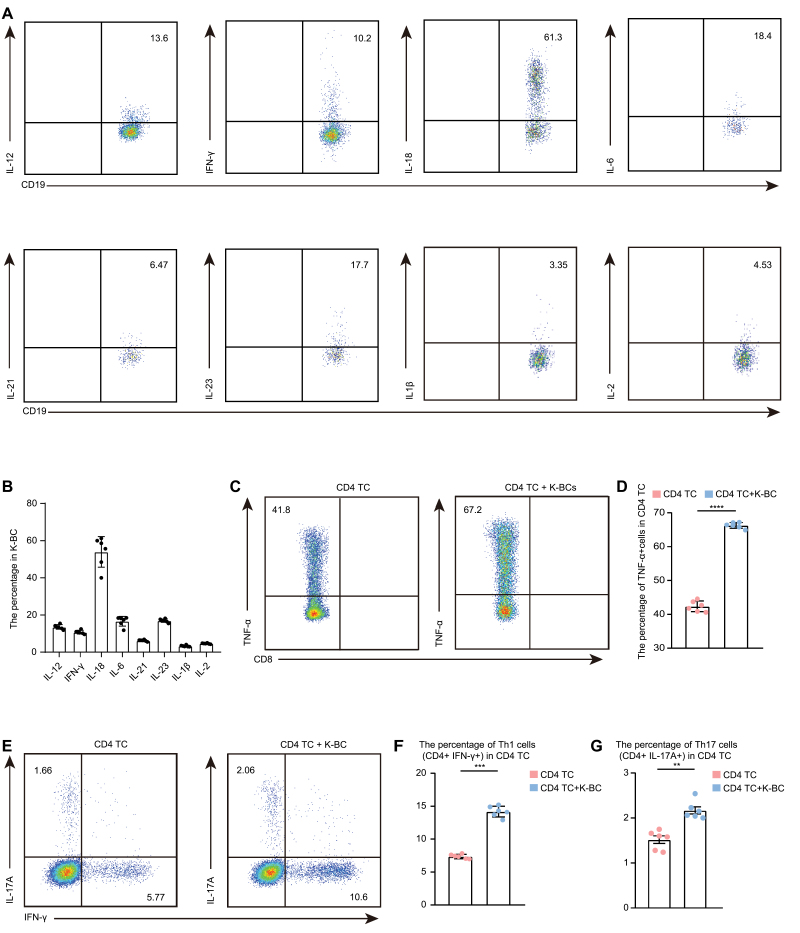
**K-BC promote the differentiation of pathogenic T cells.** (A and B) The percentage of K-BC in VKH patients expressing IL-12, IFN-γ, IL-18, IL-6, IL-21, IL-23, IL-1β, and IL-2 were measured by flow cytometry (A) and quantified (B). Each group contains six samples. The values represent the mean ± SD. (C–G) K-BC isolated from VKH patients were cocultured with CD4 TC isolated from VKH patient for 72 h. The percentage of TNF-α+ cell, Th1 (CD4^+^ IFNγ^+^), and Th17 (CD4^+^ IL-17A^+^) in CD4 TC was measured by flow cytometry (C and E) and quantified (D, F, G). Each group contains six samples. The values represent the mean ± SD. Significance was determined using unpaired Student’s *t*-test. **P < 0.01, ***P < 0.001, ****P < 0.0001.

T helper (Th)17 and Th1 cells are the dominant pathogenesis-related cells in uveitis, as reported in previous studies [13, 14]. Numerous studies also indicated their involvement in VKH disease and other autoimmune diseases [15, 16, 38, 39]. In our scRNA-seq data, GO analysis indicated that the Th17 cell differentiation pathway and IFN-γ cytokine pathway related to Th1 cell differentiation were upregulated in NoBCs in VKH disease, suggesting the assisting potential of K-BC in the differentiation in Th1 and Th17 cells (Fig. 2L). Thus, to identify the possible function of K-BCs in VKH disease, we evaluated the expression of cytokines related to Th1 cell differentiation (IL-12, IFN-γ, and IL-18) [40, 41], Th17 cell differentiation (IL-6, IL-21, IL-23, and IL-1β) [42, 43], and general cell proliferation and differentiation (IL-2) [44] by K-BCs from VKH patients. As shown by the flow cytometry results, an array of cytokines could be produced by K-BCs, including IL-12, IL-18, IFN-γ, IL-1β, IL-2, IL-4, IL-6, IL-21, and IL-23 (Figs. 5A, 5B and S7C). In addition, co-culture of isolated K-BCs with CD4^+^ TCs contributed to an increase in the proportion of CD4^+^ TNF-α^+^ cells, CD4^+^ IL-17^+^ cells (Th17), and CD4^+^ IFN-γ^+^ cells (Th1) (Fig. 5C–5G). These results indicated that K-BCs could promote the differentiation of pathogenic TCs and induce inflammation, thus may contribute to VKH disease.

## Discussion

**Figure 6.**
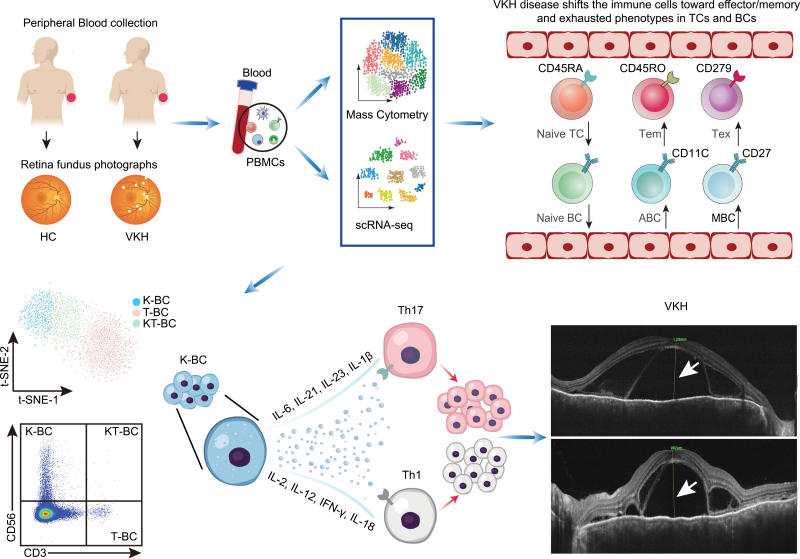
**Peripheral immune cell atlas and function of K-BCs in VKH disease.** A mixture of inflammation, effector, and exhausted states in VKH disease. Natural K-BC characterized by expressing CD19 and CD56, might be correlated with VKH disease by promoting the differentiation of pathogenic T cells.

VKH disease is a severe autoimmune disease that is a major cause of blindness. Here, we present the first comprehensive single-cell analysis of circulating immune cells in patients with VKH disease, showing specific disease-associated alterations in immune cell composition and gene expression. Prominent upregulation of inflammatory gene expression, as well as a shift toward effector/memory and exhausted phenotypes in the composition of TCs and BCs, was indicated using scRNA-seq and CyTOF data. Intriguingly, we recognized a newly identified B cell subset, K-BCs, in our data. Their role in autoimmune disease has not been demonstrated. Our study is the first to reveal that K-BCs might be associated with the pathogenesis of VKH disease and serve as an indicator of disease severity and treatment response (Fig. 6).

The mechanism of VKH disease is still unclear, which is related to the limitations of previous approaches. With the development of single-cell analysis, research is no longer limited to certain cells or biological factors and analysis of a large number of cell types at one time is possible [45]. Hu et al. performed scRNA-seq in VKH disease, but their study focused only on isolated circulating MC [19]. Therefore, it is necessary to establish a comprehensive atlas of the peripheral immune system in patients with VKH disease. Our study combined scRNA-seq and CyTOF to delineate changes in gene expression and immune cell composition in VKH disease. At the transcriptional level, our data indicated an inflammatory and activated state in VKH disease. A previous study suggested that activated antigen-specific TCs contribute to the autoimmune response in VKH disease [46, 47]. In our study, TCs showed the highest number of DEGs in VKH disease compared with the other major immune cell types. Several inflammation- or cell activation-related genes were upregulated commonly in CD4^+^ and CD8^+^ TC subsets. These results provide additional evidence for the active involvement of TCs in VKH disease pathogenesis. According to innate immune cells, MCs are thought to be an important group of innate immune cells mediating inflammation and tissue injury in autoimmune conditions [48]. MCs in our study also exhibited an evident pro-inflammation phenotype compared to DCs and NKs. Additionally, MC subsets, as well as cDC2, exhibited upregulated antigen processing and presentation-related gene expression, indicating their involvement in the adaptive immune response in VKH disease. At the protein level, our study identified a shift from naïve to effector/memory and exhausted phenotypes in the composition of TCs and BCs in patients with VKH disease. Naïve lymphocytes are activated by antigens and differentiate into effector/memory cells [49]. TC exhaustion is associated with persistent antigen stimulation and inflammation [50]. Therefore, our CyTOF data at protein level was consistent with the scRNA-seq data at transcriptional level and cooperatively indicated inflammatory, effector, and exhausted states of the peripheral immune system of patients with VKH disease.

As a crucial component of the adaptive immune system, BCs have a wide range of effector functions including antibody secretion, antigen presentation, cytokine production, and immune memory generation [51]. More recently, numerous studies on BC deletion therapy have extended the role of BCs to a broad range of autoimmune diseases such as systemic lupus erythematosus and multiple sclerosis [52–54]. Although BCs contribute to autoimmunity, the exact transcriptional signatures, alterations in gene expression, and cellular compositional changes in autoimmune conditions remain unclear. BCs in the human blood are conventionally divided into naïve, memory, age-related, and plasma cells based on their transcriptome [55]. We also observed the corresponding BC subsets in our scRNA-seq and CyTOF data. NBCs, MBCs, and ABCs exhibited enhanced cell activation, antigen processing, and presentation in VKH disease. Notably, we recognized NoBCs, which were not included in classical BC subsets. NoBCs exhibited upregulation of genes involved in pathways related to pathogenic TC differentiation in VKH disease. Using scRNA-seq and flow cytometry, we identified and confirmed the heterogeneity of NoBCs. NoBCs incorporated K-BC (CD19^+^ CD56^+^ BC), T-BC (CD19^+^ CD3^+^ BC), and KT-BC (CD19^+^ CD56^+^ CD3^+^ BC). The phenotypes of K-BCs and T-BCs have been reported previously [33, 35–37] and the relatively rare KT-BCs were reported in our study. Among them, K-BCs are found in both human and mouse and play a role in protection against infection [33, 35, 36]. Liu et al. further reported their relationship with hepatocellular carcinoma [36]. Our study is the first to extend their role in VKH disease, an autoimmune-related disease.

In our study, K-BCs increased in VKH disease, and more importantly, their proportion decreased after treatment and disease improvement. Thus, the percentage of K-BC may serve as a biomarker for monitoring VKH disease severity and treatment response. Further experiments are warranted to elucidate the function of K-BCs. Previous studies have reported that K-BCs mainly exert their anti-infection effects by secreting IL-12 and IL-18 [33, 35] through which K-BCs activate NK group I innate lymphoid cells. Our experiments showed that K-BCs produced a series of Th17 differentiation-promoting cytokines (IL-6, IL-21, IL-23, and IL-1β) [40, 41] and Th1 differentiation-promoting cytokines (IL-12, IFN-γ, and IL-18) [42, 43]. Notably, our results confirmed that K-BCs promoted the differentiation of Th1 and Th17 cells by co-culture experiment. Th17 and Th1 cells play pathogenic roles in several autoimmune diseases including uveitis [14, 56, 57]. These results indicate that K-BCs might be involved in the pathogenesis of VKH disease and even a wider range of autoimmune diseases via promoting pathogenic TCs. Although clinical examinations, such as OCT, can be used to evaluate VKH disease severity and treatment efficacy, these measurements provide little information about the pathogenesis, immune responses, and mechanisms following treatment. Our study provides evidence that K-BCs may serve as a pathologically significant blood-based index for VKH disease.

In summary, our study provides the first comprehensive peripheral immune atlas of VKH disease at single-cell resolution. Our study implicated that K-BCs could act as a biomarker to evaluate the disease severity and treatment response in patients with VKH disease. Moreover, K-BCs have been validated to promote the differentiation of pathogenic TCs, and thus, may be involved in the pathogenesis of VKH disease and even a broader range of autoimmune diseases.

## Research limitations

This study also has several limitations. VKH disease is an eye disease, but our study can only explore its mechanism through PBMCs. Due to the lack of effective ways to obtain the eyes of patients with VKH, our study cannot explore its mechanism *in situ* lesions. Admittedly, our study has the limitation of a low sample capacity, we will expand the sample size in our further investigation.

## Materials and methods

### Human subjects

The patients with VKH disease were enrolled in the Zhongshan Ophthalmic Center, Sun Yat-sen University, Guangzhou, China. These patients showed initial active uveitis without any treatment before blood drawing and were diagnosed based on the disease manifestations, and the results of standard coherent optical tomography, and indocyanine green fluorescein angiography, according to the Revised Diagnostic Criteria (RDC) of VKH Disease [5]. Written informed consent was acquired from all subjects. We collected blood samples from: 8 subjects diagnosed with VKH and 8 HC in Cohort-1; 10 patients with VKH disease, and 5 HC in Cohort-2. Individuals with comorbidities including diabetes, hypertension, cancer, and steroid usage were excluded. No remarkable gender or age differences were reported in the cohorts.

For immunosuppressive therapy: patients with VKH disease received oral corticosteroid treatment with an initial dose of 80–100 mg/d and maintained for 2 weeks. Then, oral corticosteroid treatment was tapered off gradually. After 1 month of treatment with systemic corticosteroids, no evidence of acute ocular inflammation, including choroiditis, serous retinal detachment, disk edema, or exudative retinal detachment, was observed by clinical examination. Blood samples were obtained after 1- and 3-month treatment, respectively.

All protocols were reviewed and authorized by the Medical Ethics Committee of the Guangzhou Zhongshan Ophthalmic Center (ID: 2020KYPJ124).

### Isolation of PBMCs

We extracted the venous blood samples from all donors using Ficoll-Hypaque solution (GE Healthcare, PA, USA), followed by heparinization of the blood, then processing via standard density gradient centrifugation approaches to obtain PBMCs. Trypan Blue was employed to determine the PBMCs viability, as well as the quantity in single-cell suspensions. The cell viability was >90% in all the samples.

### Flow cytometry assay

PBMCs were obtained and suspended in phosphate buffered saline (PBS). After staining using the Fixable Viability Dye from Zombie NIR™ Fixable Viability Kit (Thermo Fisher) or LIVE/DEAD™ Fixable Yellow Dead Cell Stain Kit (Biolegend) for 30 min on 4°C, we rinsed the cells using 1 mL PBS enriched with 2% Fetal Bovine Serum (FBS, GIBCO, Grand Island, NY, USA). Afterwards, the cells were stained with surface markers for 30 min on 4°C and analysis by flow cytometer (BD LSRFortessa). For intracellular staining, following surface marker staining, we stimulated the cells by phorbol myristate acetate (PMA, 5 ng/mL, Sigma), ionomycin (500 ng/mL, Sigma), as well as brefeldin A (BFA, 1 µg/mL, Sigma) for 6 h, followed by fixation and permeabilization, and subjected to incubation with antibodies against intracellular antigens. The FlowJo (version 10.0.7, Tree Star, Ashland, OR, USA) was employed to assess the results.

### Co-culture of CD4^+^ T cell with K-BC

CD4^+^ T cell (CD3^+^ CD4^+^) and K-BC (CD19^+^ CD56^+^) were sorted from PBMCs of the same VKH patient via a flow cytometer (FACS Aria III, BD) for further assessments. Purity of CD4^+^ T cell and K-BC were both over 95%, which was established via post sort evaluation of flow cytometry.

Sorted K-BC co-culture with CD4^+^ T cells at a 1:10 K-BC: T cell ratio in the presence or absence of 20 nM Tofacitinib (Selleck) for 72 h. Following the exposure to PMA, ionomycin and BFA for 6 h, intracellular IFN-γ, TNF-α, and IL-17 of CD4 cells were explored via flow cytometry.

### scRNA-seq pre-processing

Conversion of the suspensions consisting of single-cells into sequencing libraries of barcoded scRNA using the Chromium Single Cell 5 v2 Reagent Kit (10× Genomics, Pleasanton, CA, USA; 120237) as per the instructions of the manufacturer. Sequencing was accomplished on the 10× Genomics chromium platform Illumina NovaSeq6000 with the Chromium Single Cell 5ʹLibrary, Gel Bead and Multiplex Kit, and Chip Kit (10× Genomics). Moreover, the FastQC software was employed to perform quality checks. The CellRanger software was employed in the preliminary preparation of the sequencing data.

The command cell ranger count in Cell Ranger v3.0.2 (10× Genomics) was employed in de-multiplexing the off-machine 5ʹ-expression sequencing data and aligned to the human transcriptome (build GRCh38). Pooling of the generated outputs of the samples was done to obtain a combined raw expression pattern (gene numbers versus cells) using the function cellranger aggr. Then, the data were filtered, and then normalized (their dimensionality was diminished), followed by clustering. Afterwards, differential gene expression evaluation was conducted after computation of the single-cell expression pattern by Scanpy (version 1.4.6) implemented in Python (version 3.7.7). The data collection, as well as the successive synthesis were conducted in an unsupervised approach, but not blinded.

### scRNA-seq quality control

In the quality control, the cell populations that were filtered were primarily those expressing HBA1, HBB, and numerous lights, as well as heavy chain transcripts, that were uncovered as the population of cells contaminated with RBCs. Similarly, numerous cluster-expressing genes with no significance (*P* ≥ 0.1), as per the 10× Genomics Loupe Cell Browser with the default algorithm, were removed. Of note, the *P* values were adjusted via the Benjamini-Hochberg correction for multiple tests. Importantly, we employed the run_harmony function (in pyharmony package, version 1.0.7), as well as the combat function (in Scanpy) to address the batch effect issues. Overall, 172,525 high-quality cells (HC 88,934 cells, VKH 83,591 cells) were used to further analysis after quality control.

### scRNA-seq cell clustering

We employed the sc.pp.normalize_total function to normalized the data (1 + counts per 10,000) before clustering. Afterwards, 2-dimensional *t*-SNE algorithm clustering was performed with the first 50 principal components. This was conducted following PCA on the top 5000 most variable genes using the sc.pp.highly_variable_genes function (in Scanpy) using the default settings. The Leiden clustering algorithm was employed to perform the dimensionality approach, as well as determine the remarkable clusters. The sc.tl.rank_genes_groups function (in Scanpy) under the default settings was employed to identify the signature genes for every significant cluster.

### scRNA-seq differential expression assessment

Differential expression analysis genes (DEGs) for each kind of cell (in HC versus VKH groups) was carried out though the *t*-test via the the sc.tl. rank_genes_groups function in the Scanpy package. The DEGs were defined for each cluster via the *t*-test and identified in comparison to all the other cells. A disease-linked DEG was created (adjusted *P* value < 0.05, |Log_2_FC| > 0.25) after the determination of DEGs between the HC and VKH groups.

### Gene ontology analysis

DEGs gene ontology, as well as pathway enrichment assessment, were performed and the functional profiles of the gene clusters visualized using the metascape web application [58]. The *P* values of GO terms are calculated based on the accumulative hypergeometric distribution by Metascape webtool. Consequently, we visualized 2-8 GO terms linked to disease from the top 50 abundant GO terms across the diverse kinds of cells. The gene expression pattern cluster plots, as well as the heatmaps were generated in the heatmap R package (v. 1.0.12).

### CyTOF processing and quality control

We labeled the peripheral venous blood derived cells using a unique barcode via incubation with the CD45 antibodies labeled with specific metal isotopes (162Dy, 165 426 Ho, 169Tm, 175Lu). The samples were rinsed two times using the cell staining medium (CSM, Fluidigm, Shanghai, China), followed by pooling into a single reaction tube for additional staining steps. A Maxpar Direct Immune Profiling Assay Kit (Fluidigm, South San Francisco, CA, USA) was employed in the cell surface staining.

Prior to acquiring the data, we rinsed the samples once using PBS, then using de-ionized water once, followed by re-suspension at quantities of 1.0 × 10^6^ cells/mL in a 1:10 dilution of EQ 4 Element Beads solution (Fluidigm). We acquired the samples on a Helios (Fluidigm) at 300–500 events/s under noise deduction.

Quality control: Prior to subsequent assessments, deconvolution of the barcodes was done via Cytobank uisng the manually Boolean gates in the event of samples barcoded with CD45 [59]. Gating of the data was performed to determine the cell events (DNAhi) and exclude the dying or dead cells (cisplatin+). We left the live cells for downstream clustering, as well as high dimensional assessments.

### CyTOF reduction and clustering

The datasets for CyTOF were created for the evaluation of each kind of cell. Hence, down-sampled datasets were generated constituting 95,316 TC, 35,254 NK, 22,042 BC, 39,144 MC, and 8244 DC in Cohort-1 for evaluation.

The FlowCore was employed to read, as well as process the FCS files for additional assessment. For samples with >20,000 cells, 20,000 cells were selected randomly for equal representation of the samples. Finally, the *t*-SNE dimensionality reduction algorithm was run on an integrated sample of the data in the package Seurat that is centered on harmony embedding (version 1.0.0).

### Statistical analysis

The GraphPad Prism Software (version 8.0.2, GraphPad Software) was employed for data analyses and graphics production. Statistical analysis was performed using an unpaired Student’s *t*-test and ANOVA. *P* < 0.05 were considered to be statistically significant. Detailed descriptions of statistical tests are speciﬁed in the results section and in the figure legends.

### Data availability

The single-cell sequencing data have been deposited at Genome Sequence Archive (GSA, https://ngdc.cncb.ac.cn/gsa-human/) with the project number PRJCA004235 and GSA accession number HRA000584.

## Supplementary Material

lnac047_suppl_Supplementary_Material

lnac047_suppl_Supplementary_Table_S1
