# Supplementary material for: Peripheral immune landscape and natural killer-like B cells in human Vogt-Koyanagi-Harada disease
Source: Life Med. 2022 Dec 8;1(3):387–400. doi: 10.1093/lifemedi/lnac047 (PMC11749541; doi:10.1093/lifemedi/lnac047)
Supplement: lnac047_suppl_Supplementary_Material [file lnac047_suppl_Supplementary_Material.pdf]

Supplemental Information for "Peripheral immune landscape and natural killer-like B cells in human Vogt-Koyanagi-Harada disease"

Figure S1. Clusters of major immune cell types originated from scRNA-seq data.

(A) Heatmap showing scaled expression of discriminative gene sets for major immune cell types originated from scRNA-seq data. (B) *t*-SNE plots of canonical markers for major immune cell types originated from scRNA-seq data.

Figure S2. Clusters of immune cell subsets originated from scRNA-seq data.

(A) *t*-SNE plots of TC subsets originated from scRNA-seq data. (B) *t*-SNE plots of canonical markers for TC subsets originated from scRNA-seq data. (C) Heatmap showing scaled expression of discriminative gene sets for TC subsets originated from scRNA-seq data. (D) *t*-SNE plots of CD4 TC subsets originated from scRNA-seq data. (E) *t*-SNE plots of canonical markers for CD4 TC subsets originated from scRNA-seq data. (F) Heatmap showing scaled expression of discriminative gene sets for CD4 TC subsets originated from scRNA-seq data. (G) *t*-SNE plots of CD8 TC subsets originated from scRNA-seq data. (H) t-SNE plots of canonical markers for CD8 TC subsets originated from scRNA-seq data. (I) Heatmap showing scaled expression of discriminative gene sets for CD8 TC subsets originated from scRNA-seq data. (J) *t*-SNE plots of NK subsets originated from scRNA-seq data. (K) *t*-SNE plots of canonical markers for NK subsets originated from scRNA-seq data. (L) Heatmap showing scaled expression of discriminative gene sets for NK subsets originated from scRNA-seq data. (M) *t*-SNE plots of monocyte subsets originated from scRNA-seq data. (N) *t*-SNE plots of canonical markers for monocyte subsets originated from scRNA-seq data. (O) Heatmap showing scaled expression of discriminative gene sets for monocyte subsets originated from scRNA-seq data. (P) *t*-SNE plots of DC subsets

originated from scRNA-seq data. (Q) *t*-SNE plots of canonical markers for DC subsets originated from scRNA-seq data. (R) Heatmap showing scaled expression of discriminative gene sets for DC subsets originated from scRNA-seq data.

Figure S3. Clusters of major immune cell types and T cell subsets originated from CyTOF data.

(A) *t*-SNE plots of PBMCs of HC and VKH originated from mass cytometry (CyTOF) data. (B) *t*-SNE plots of canonical markers for major immune cell types originated from CyTOF data. (C) *t*-SNE plots of T cell subsets originated from CyTOF data. (D) *t*-SNE plots of canonical markers for T cell subsets originated from CyTOF data.

Figure S4. Clusters of immune cell subsets originated from CyTOF data.

(A) *t*-SNE plots of NK subsets originated from CyTOF data. (B) *t*-SNE plots of canonical markers for NK subsets originated from CyTOF data. (C) *t*-SNE plots of myeloid cell subsets originated from CyTOF data. (D) *t*-SNE plots of canonical markers for myeloid cell subsets originated from CyTOF data. (E) *t*-SNE plots of B cell subsets originated from CyTOF data. (F) *t*-SNE plots of canonical markers for B cell subsets originated from CyTOF data.

Figure S5. GO analysis of DEG.

(A) Heatmap showing representative GO terms enriched in downregulated DEGs of B cell subsets in the VKH/HC comparison group. (B) Heatmap showing representative GO terms enriched in upregulated DEGs of NK, MC, and DC in the VKH/HC comparison group. (C) Heatmap showing representative GO terms enriched in downregulated DEGs of NK, MC, and DC in the VKH/HC comparison group. (D) Bar plot showing representative GO terms enriched in up- and down-regulated DEGs of NK subsets in the VKH/HC comparison group. (E) Bar plot showing representative GO terms enriched in up- and down-regulated DEGs of MC subsets in the VKH/HC

comparison group. (F) Bar plot showing representative GO terms enriched in up-regulated DEGs of DC subsets in the VKH/HC comparison group.

Figure S6. Percentage of immune cell subsets from CyTOF data.

(A-D) Bar chat shows percentage of TC, BC, NK, and myeloid cell in total PBMCs from HC (*n* = 10) and VKH (*n* = 5) group originated from CyTOF data. The values represent the mean ± SD. Significance was determined using unpaired student's *t* test. ns, not significant. (E-G) Bar chat shows percentage of CD4 T cell subsets in CD4 TC from HC (*n* = 10) and VKH (*n* = 5) group originated from CyTOF data. The values represent the mean ± SD. Significance was determined using unpaired student's *t* test. ns, not significant. (H-J) Bar chat shows percentage of CD8 T cell subsets in CD8 TC from HC (*n* = 10) and VKH (*n* = 5) group originated from CyTOF data. The values represent the mean ± SD. Significance was determined using unpaired student's *t* test. ns, not significant. (K-L) Bar chat shows percentage of B cell subsets in BC from HC (*n* = 10) and VKH (*n* = 5) group originated from CyTOF data. The values represent the mean ± SD. Significance was determined using unpaired student's *t* test. ns, not significant. (M-O) Bar chat shows percentage of MC subsets in total PBMCs from HC (*n* = 10) and VKH (*n* = 5) group originated from CyTOF data. The values represent the mean ± SD. Significance was determined using unpaired student's *t* test. ns, not significant. (P-Q) Bar chat shows percentage of DC subsets in total PBMCs from HC (*n* = 10) and VKH (*n* = 5) group originated from CyTOF data. The values represent the mean ± SD. Significance was determined using unpaired student's *t* test. ns, not significant. (R-T) Bar chat shows percentage of NK subsets in total PBMCs from HC (*n* = 10) and VKH (*n* = 5) group originated from CyTOF data. The values represent the mean ± SD. Significance was determined using unpaired student's *t* test. *$P < 0.05$. ns, not significant. (U) Gating strategy of NoBC.

Figure S7. Percentage of T-BCs and KT-BCs, and non-stimulated control of K-BCs.

(A) The percentage of T-BCs in BC from VKH patient in different stages (before treatment, after treatment for one month, and after treatment for three months). Each group contains six samples. The values represent the mean ± SD. Significance was determined using ANOVA. ns, not significant. (B) The percentage of KT-BCs in BC from VKH patient in different stages (before treatment, after treatment for one month, and after treatment for three months). Each group contains six samples. The values represent the mean ± SD. Significance was determined using ANOVA. ns, not significant. (C) Non-stimulated control of K-BCs in IL-12, IFN-γ, IL-18, IL-6, IL-21, IL-23, IL-1β, and IL-2.

Figure S1

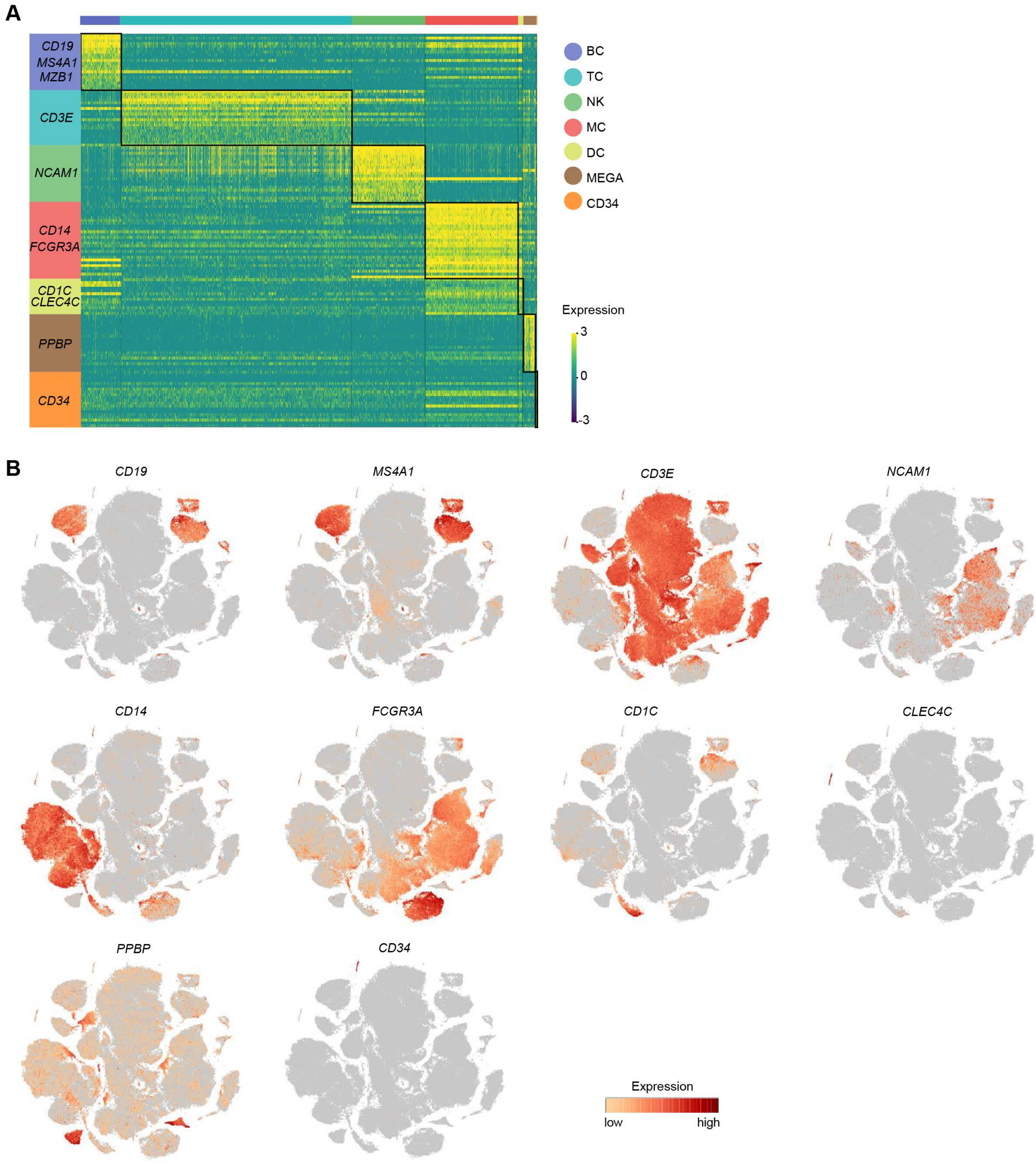
A
CD19
MS4A1
MZB1
CD3E
NCAM1
CD14
FCGR3A
CD1C
CLEC4C
PPBP
CD34
BC
TC
NK
MC
DC
MEGA
CD34
Expression
3
0
-3
B
CD19
MS4A1
CD3E
NCAM1
CD14
FCGR3A
CD1C
CLEC4C
PPBP
CD34
Expression
low
high

Figure S2

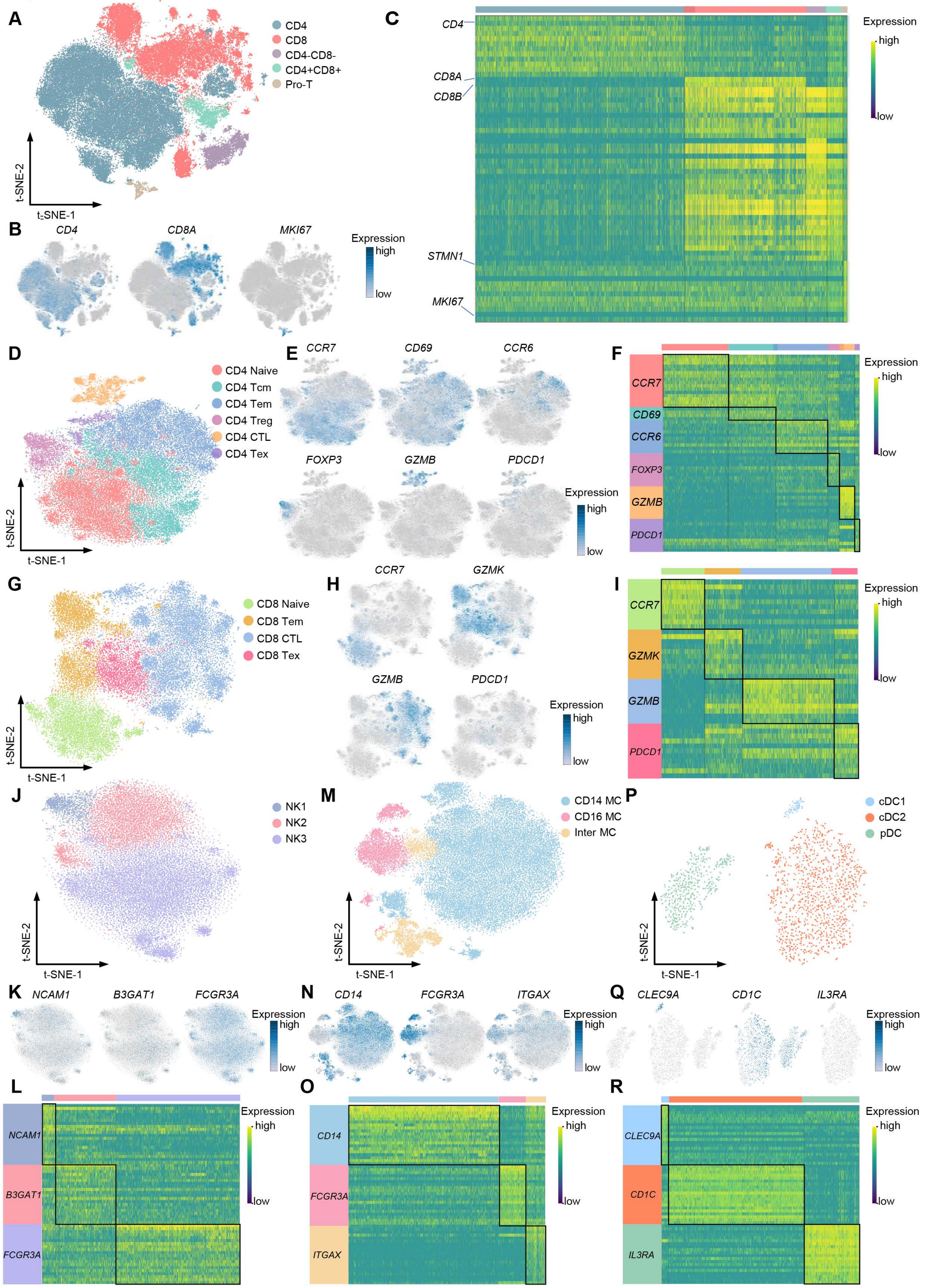

A
CD4
CD8
CD4-CD8-
CD4+CD8+
Pro-T
t-SNE-2
t-SNE-1
B
CD4
CD8A
MKI67
Expression
high
low
C
CD4
CD8A
CD8B
STMN1
MKI67
Expression
high
low
D
CD4 Naive
CD4 Tcm
CD4 Tem
CD4 Treg
CD4 CTL
CD4 Tex
t-SNE-2
t-SNE-1
E
CCR7
CD69
CCR6
FOXP3
GZMB
PDCD1
Expression
high
low
F
CCR7
CD69
CCR6
FOXP3
GZMB
PDCD1
Expression
high
low
G
CD8 Naive
CD8 Tem
CD8 CTL
CD8 Tex
t-SNE-2
t-SNE-1
H
CCR7
GZMK
GZMB
PDCD1
Expression
high
low
I
CCR7
GZMK
GZMB
PDCD1
Expression
high
low
J
NK1
NK2
NK3
t-SNE-2
t-SNE-1
M
CD14 MC
CD16 MC
Inter MC
t-SNE-2
t-SNE-1
P
cDC1
cDC2
pDC
t-SNE-2
t-SNE-1
K
NCAM1
B3GAT1
FCGR3A
Expression
high
low
N
CD14
FCGR3A
ITGAX
Expression
high
low
Q
CLEC9A
CD1C
IL3RA
Expression
high
low
L
NCAM1
B3GAT1
FCGR3A
Expression
high
low
O
CD14
FCGR3A
ITGAX
Expression
high
low
R
CLEC9A
CD1C
IL3RA
Expression
high
low

Figure S3

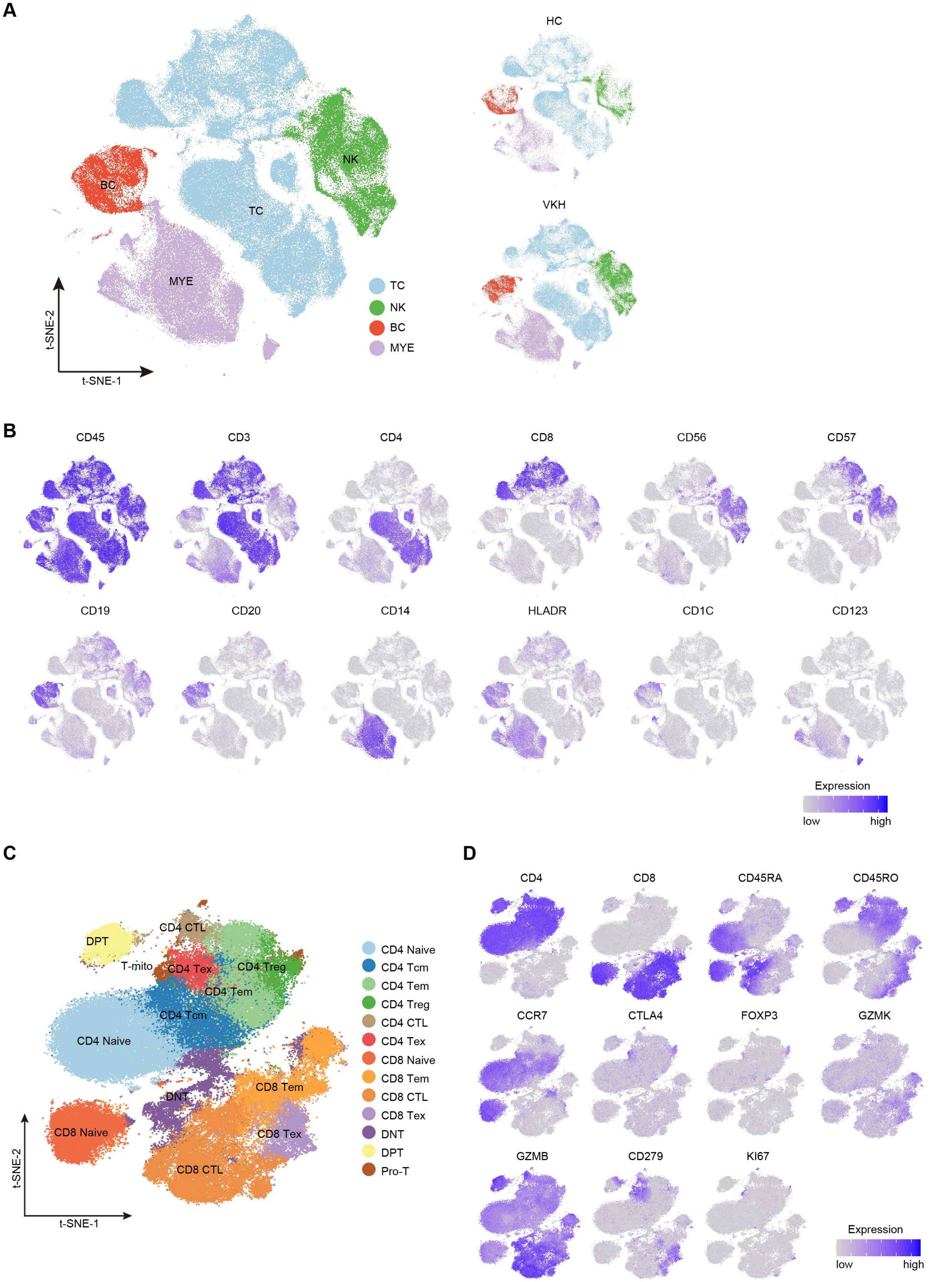
A
HC
VKH
BC
TC
NK
MYE
TC
NK
BC
MYE
t-SNE-2
t-SNE-1
B
CD45
CD3
CD4
CD8
CD56
CD57
CD19
CD20
CD14
HLADR
CD1C
CD123
Expression
low
high
C
DPT
CD4 CTL
T-mito
CD4 Tex
CD4 Treg
CD4 Tem
CD4 Tcm
CD4 Naive
DNT
CD8 Tem
CD8 Naive
CD8 Tex
CD8 CTL
t-SNE-2
t-SNE-1
CD4 Naive
CD4 Tcm
CD4 Tem
CD4 Treg
CD4 CTL
CD4 Tex
CD8 Naive
CD8 Tem
CD8 CTL
CD8 Tex
DNT
DPT
Pro-T
D
CD4
CD8
CD45RA
CD45RO
CCR7
CTLA4
FOXP3
GZMK
GZMB
CD279
KI67
Expression
low
high

Figure S4

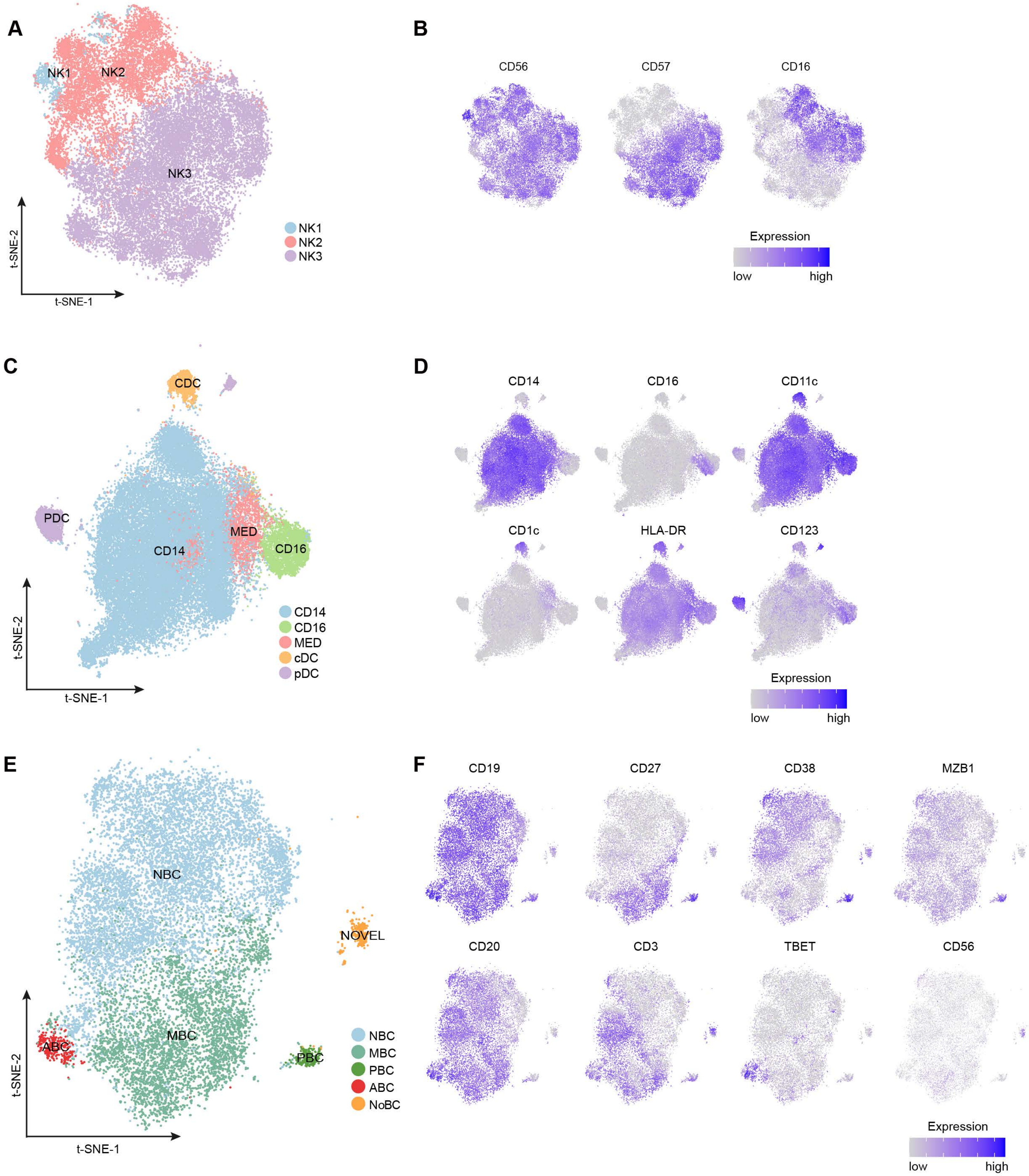
A
NK1
NK2
NK3
t-SNE-2
t-SNE-1
NK1
NK2
NK3
B
CD56
CD57
CD16
Expression
low
high
C
CDC
PDC
CD14
MED
CD16
t-SNE-2
t-SNE-1
CD14
CD16
MED
cDC
pDC
D
CD14
CD16
CD11c
CD1c
HLA-DR
CD123
Expression
low
high
E
NBC
NOVEL
ABC
MBC
PBC
t-SNE-2
t-SNE-1
NBC
MBC
PBC
ABC
NoBC
F
CD19
CD27
CD38
MZB1
CD20
CD3
TBET
CD56
Expression
low
high

Figure S5

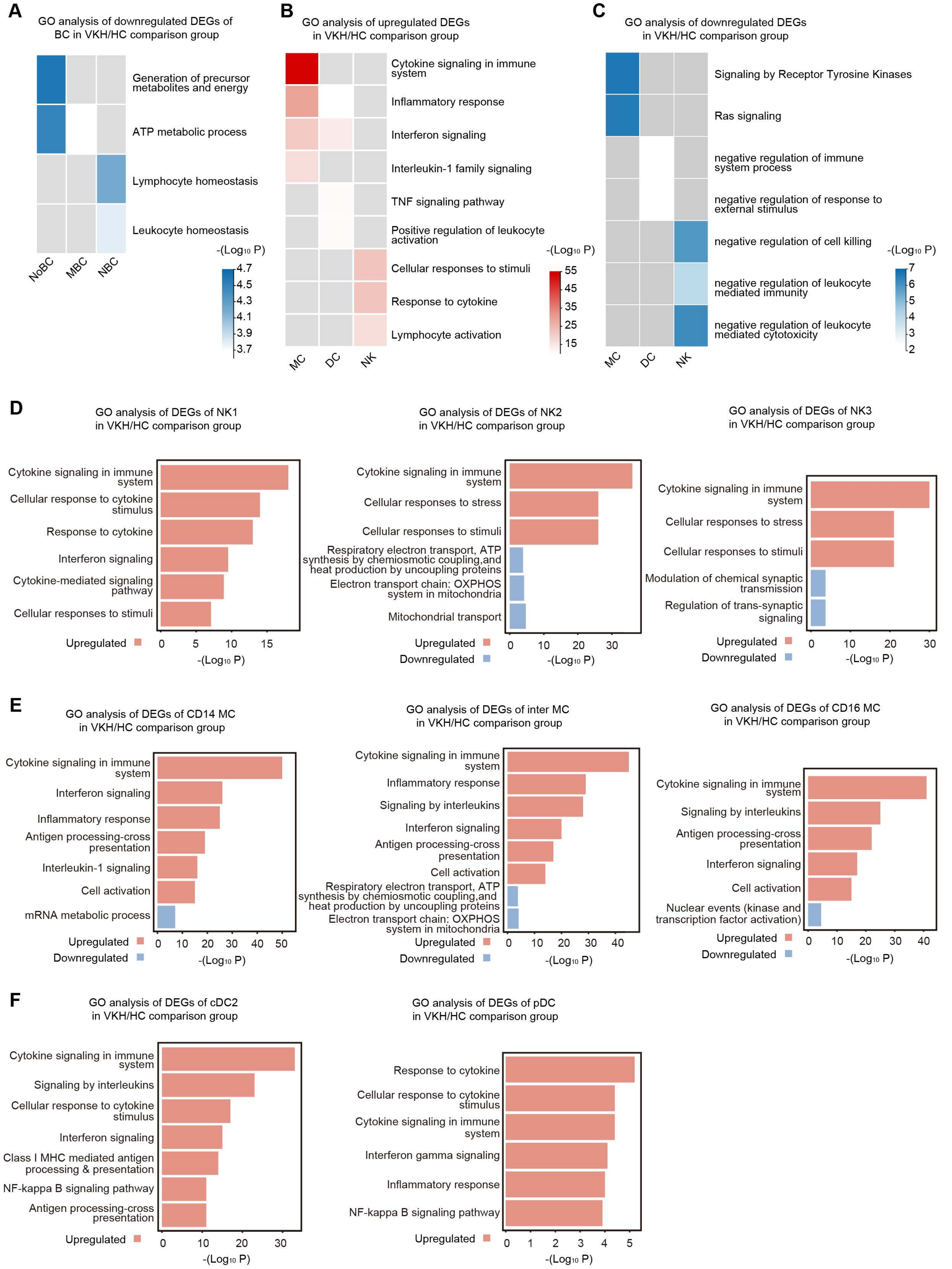
A
GO analysis of downregulated DEGs of BC in VKH/HC comparison group
Generation of precursor metabolites and energy
ATP metabolic process
Lymphocyte homeostasis
Leukocyte homeostasis
NoBC
MBC
NBC
-(Log10 P)
4.7
4.5
4.3
4.1
3.9
3.7
B
GO analysis of upregulated DEGs in VKH/HC comparison group
Cytokine signaling in immune system
Inflammatory response
Interferon signaling
Interleukin-1 family signaling
TNF signaling pathway
Positive regulation of leukocyte activation
Cellular responses to stimuli
Response to cytokine
Lymphocyte activation
MC
DC
NK
-(Log10 P)
55
45
35
25
15
C
GO analysis of downregulated DEGs in VKH/HC comparison group
Signaling by Receptor Tyrosine Kinases
Ras signaling
negative regulation of immune system process
negative regulation of response to external stimulus
negative regulation of cell killing
negative regulation of leukocyte mediated immunity
negative regulation of leukocyte mediated cytotoxicity
MC
DC
NK
-(Log10 P)
7
6
5
4
3
2
D
GO analysis of DEGs of NK1 in VKH/HC comparison group
Cytokine signaling in immune system
Cellular response to cytokine stimulus
Response to cytokine
Interferon signaling
Cytokine-mediated signaling pathway
Cellular responses to stimuli
Upregulated
0 5 10 15
-(Log10 P)
GO analysis of DEGs of NK2 in VKH/HC comparison group
Cytokine signaling in immune system
Cellular responses to stress
Cellular responses to stimuli
Respiratory electron transport, ATP synthesis by chemiosmotic coupling,and heat production by uncoupling proteins
Electron transport chain: OXPHOS system in mitochondria
Mitochondrial transport
Upregulated
Downregulated
0 10 20 30
-(Log10 P)
GO analysis of DEGs of NK3 in VKH/HC comparison group
Cytokine signaling in immune system
Cellular responses to stress
Cellular responses to stimuli
Modulation of chemical synaptic transmission
Regulation of trans-synaptic signaling
Upregulated
Downregulated
0 10 20 30
-(Log10 P)
E
GO analysis of DEGs of CD14 MC in VKH/HC comparison group
Cytokine signaling in immune system
Interferon signaling
Inflammatory response
Antigen processing-cross presentation
Interleukin-1 signaling
Cell activation
mRNA metabolic process
Upregulated
Downregulated
0 10 20 30 40 50
-(Log10 P)
GO analysis of DEGs of inter MC in VKH/HC comparison group
Cytokine signaling in immune system
Inflammatory response
Signaling by interleukins
Interferon signaling
Antigen processing-cross presentation
Cell activation
Respiratory electron transport, ATP synthesis by chemiosmotic coupling,and heat production by uncoupling proteins
Electron transport chain: OXPHOS system in mitochondria
Upregulated
Downregulated
0 10 20 30 40
-(Log10 P)
GO analysis of DEGs of CD16 MC in VKH/HC comparison group
Cytokine signaling in immune system
Signaling by interleukins
Antigen processing-cross presentation
Interferon signaling
Cell activation
Nuclear events (kinase and transcription factor activation)
Upregulated
Downregulated
0 10 20 30 40
-(Log10 P)
F
GO analysis of DEGs of cDC2 in VKH/HC comparison group
Cytokine signaling in immune system
Signaling by interleukins
Cellular response to cytokine stimulus
Interferon signaling
Class I MHC mediated antigen processing & presentation
NF-kappa B signaling pathway
Antigen processing-cross presentation
Upregulated
0 10 20 30
-(Log10 P)
GO analysis of DEGs of pDC in VKH/HC comparison group
Response to cytokine
Cellular response to cytokine stimulus
Cytokine signaling in immune system
Interferon gamma signaling
Inflammatory response
NF-kappa B signaling pathway
Upregulated
0 1 2 3 4 5
-(Log10 P)

Figure S6

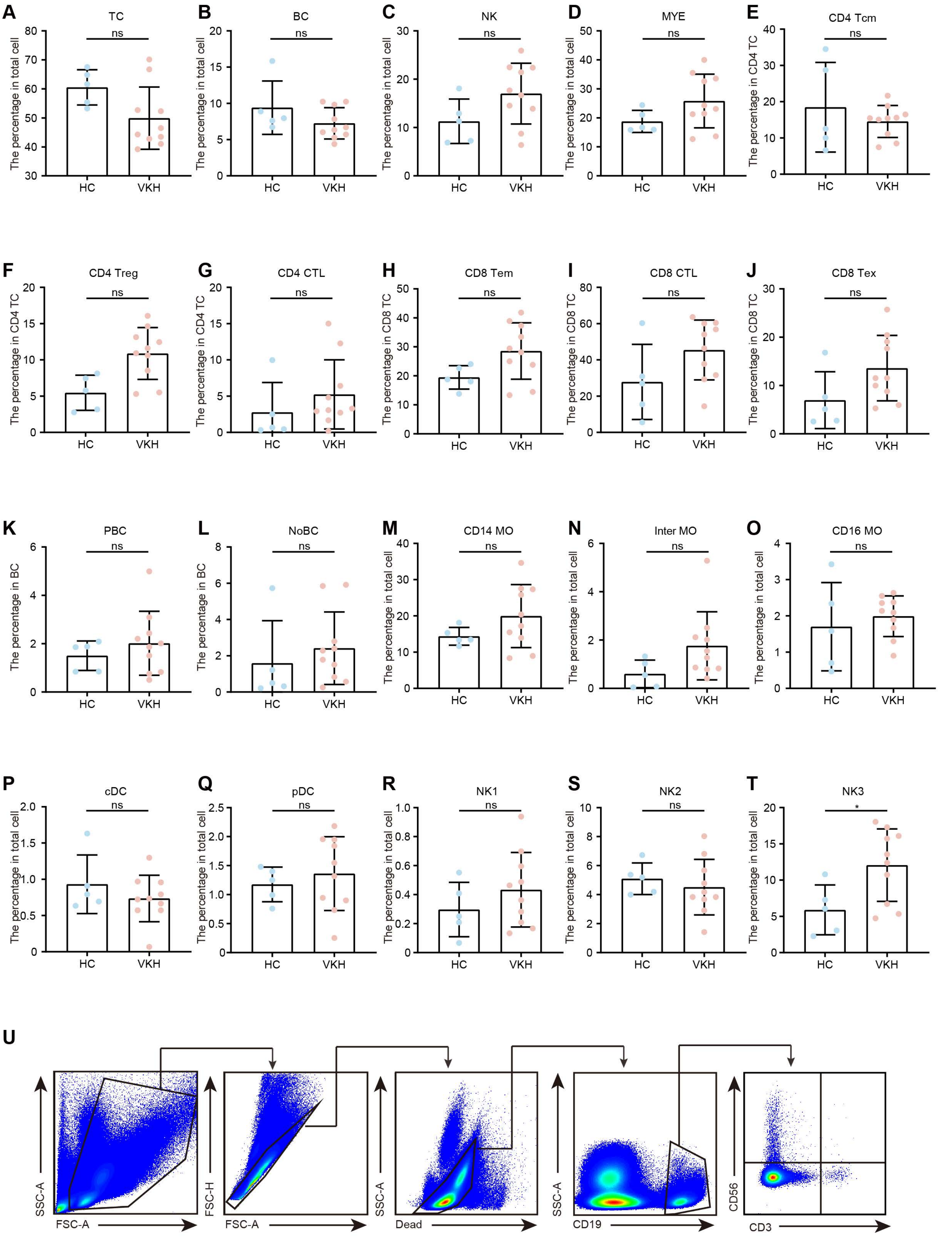

A
TC
B
BC
C
NK
D
MYE
E
CD4 Tcm
F
CD4 Treg
G
CD4 CTL
H
CD8 Tem
I
CD8 CTL
J
CD8 Tex
K
PBC
L
NoBC
M
CD14 MO
N
Inter MO
O
CD16 MO
P
cDC
Q
pDC
R
NK1
S
NK2
T
NK3
ns
*
HC
VKH
The percentage in total cell
The percentage in CD4 TC
The percentage in CD8 TC
The percentage in BC
U
SSC-A
FSC-A
FSC-H
Dead
CD19
CD56
CD3

Figure S7

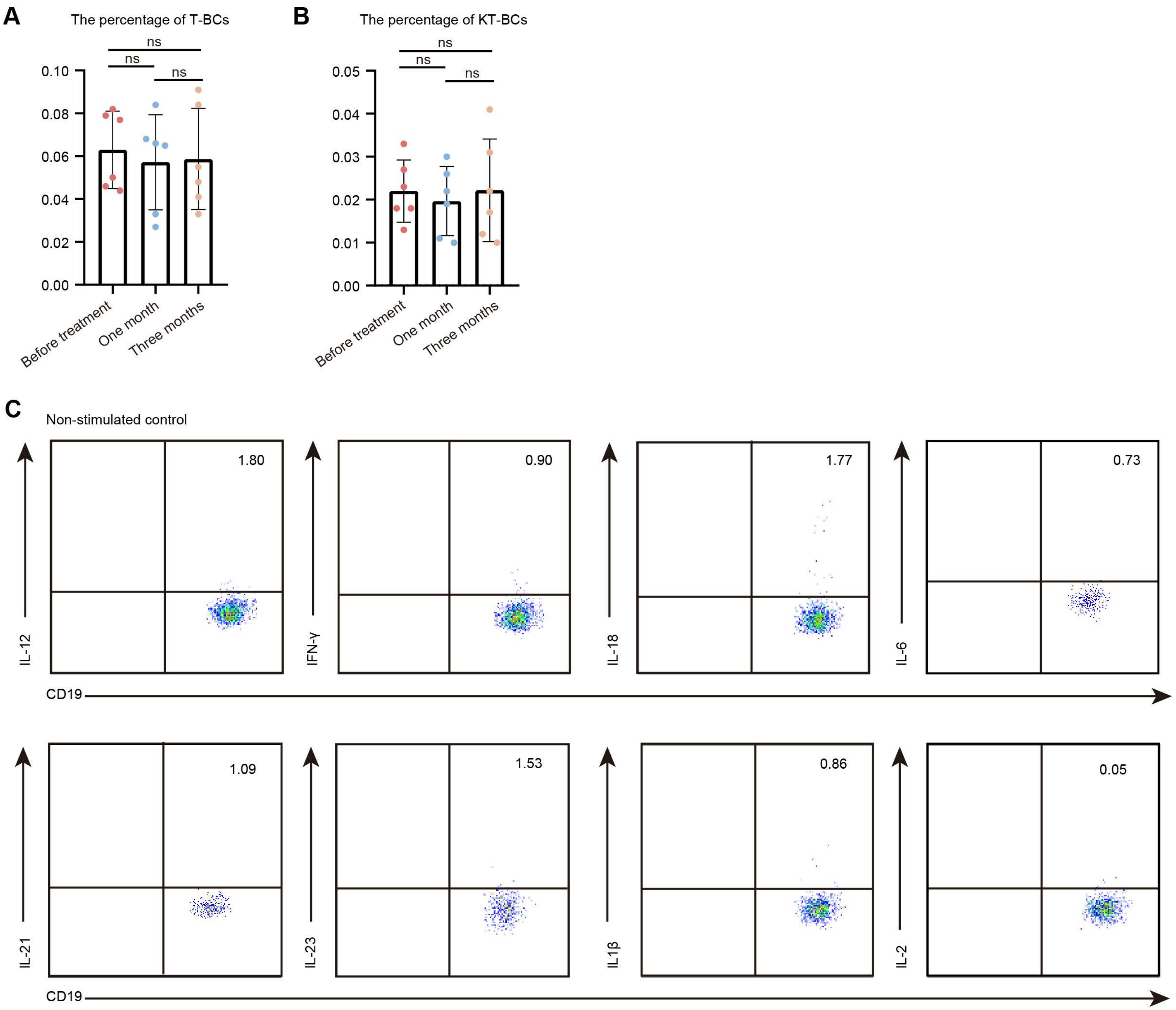
A
The percentage of T-BCs
ns
ns
ns
0.10
0.08
0.06
0.04
0.02
0.00
Before treatment
One month
Three months
B
The percentage of KT-BCs
ns
ns
ns
0.05
0.04
0.03
0.02
0.01
0.00
Before treatment
One month
Three months
C
Non-stimulated control
IL-12
1.80
IFN-γ
0.90
IL-18
1.77
IL-6
0.73
CD19
IL-21
1.09
IL-23
1.53
IL1β
0.86
IL-2
0.05
CD19
